# Psychological stress induced zinc accumulation and up-regulation of ZIP14 and metallothionein in rat liver

**DOI:** 10.1186/1471-230X-14-32

**Published:** 2014-02-18

**Authors:** Xue Tian, Yuanyuan Zheng, Yingjie Li, Zhilei Shen, Liping Tao, Xiao Dou, Jianxin Qian, Hui Shen

**Affiliations:** 1 Department of Naval Hygiene, Second Military Medical University, 800 Xiangyin Road, Shanghai 200433, PR China; 2 Department of Immunology, Second Military Medical University, No. 800 Xiangyin Road, Shanghai 200433, China; 3 Department of Medical Oncology, Changzheng Hospital, No. 64 Hetian Road, Shanghai 200070, China

**Keywords:** Psychological stress, Zinc, ZIP14, Metallothionein, Liver, Corticosterone

## Abstract

**Background:**

Zinc is necessary for normal liver function; and vice versa, the liver plays a central role in zinc homeostasis. The aim of present study is to assess the effects of repeated psychological stress (PS) on the zinc metabolism and related mechanism involved in zinc homeostasis in rat liver.

**Methods:**

In present study, we used communication box to create PS model and investigated the serum corticosterone (CORT), zinc level in serum and liver, liver metallothionein (MT) content and ZRT/IRT-like Protein 14 (ZIP14) mRNA expression.

**Results:**

The results showed that the serum CORT level increased and serum zinc level decreased significantly after 7 d and 14 d PS treatment. Meanwhile, zinc and MT contents in liver were elevated after 14 d PS exposure, while those in 7 d PS exposure group did not change. ZIP14 mRNA was expressed markedly at 7 d after the onset of PS, while Zip14 mRNA expression in the liver after 14 d PS exposure reached normal level compared with control group.

**Conclusions:**

The results suggest that PS exposure could induce hypozincemia, which might be related to liver zinc accumulation because of high level of MT through glucocorticoid-mediated MT synthesis and ZIP14 expression induced by interleukin-6.

## Background

Zinc plays critical roles as a co-factor in numerous transcription factors and in a wide variety of biochemical processes and functions for over 300 different enzymes. It is an essential nutrient necessary for various biochemical and physiological functions, including growth, development, and sexual maturity in males and so on. Zinc deficiency results in growth retardation, immune dysfunctions, and cognitive impairment [1]. Zinc is necessary for normal liver function; and vice versa, the liver plays a central role in Zn homeostasis. The liver represents a fast-exchangeable zinc pool with an important role in the metabolism of zinc and other trace elements [2]. Consequently, liver diseases affect zinc levels, whereas zinc deficiency could participate in their pathogenesis [3] and reduced hepatic zinc levels have been correlated with impaired liver function and regeneration [4]. Zinc also plays an important role in the therapy for several liver diseases and has been shown to attenuate or protect against a variety of hepatotoxins such as carbon tetrachloride, bromobenzene, and several metals [5-7]. It is also becoming clear that clinical and biochemical manifestations of zinc deficiency often occur when some stress is placed on organism. In liver disease, this stress may occur through increased gut permeability with endotoxemia, infections such as spontaneous bacterial peritonitis, or release of stress hormones [8].

Recently, psychological stress (PS) has attracted much attention because its significant negative effects can increase the risk of various diseases, including diabetes, cardiovascular neurodegenerative diseases, and aging [9-11]. It has been suggested that PS is associated with increased oxidant production and oxidative damage; thus, long-term exposure to PS may increase the risk of many diseases [9-11]. The etiology of these diseases has been linked to oxidative damage of DNA, proteins, and lipids, which are catalyzed by reactive oxygen species [12,13]. It has been shown that PS exposure strengthens the oxidative reactions within the brain and plasma of rats [14,15]. Our previous studies demonstrated that after repeated PS exposure, serum iron level decreased and iron significantly accumulated in the apical poles of villous enterocytes, liver, spleen, cerebral cortex, hippocampus, and striatum in rats [14,16,17]. More interesting, in the further study, we investigated the effect of zinc supplementation on the iron metabolism, erythropoiesis, and oxidative stress status in PS-induced rats. Compared to PS-treated rats with normal diet, the PS-treated rats with zinc supplementation showed increased serum iron, while iron contents in liver, spleen, and regional brain decreased [18]. The effect of PS on zinc metabolism is not fully characterized.

Some experiments showed that the zinc concentration in the liver was significantly higher in mice at 12 h after the onset of restraint stress than that without stress. The interleukin-6 (IL-6) protein level in the serum was transiently elevated at 3 h after the onset of restraint stress, and the IL-6-responsive zinc transporter ZRT/IRT-like Protein 14 (ZIP14) mRNA in the liver was expressed markedly at 6 h [19]. These results suggest that ZIP14 plays a major role in the mechanism responsible for accumulation of zinc in the liver under restraint stress. Zinc homeostasis is coordinated via regulation by proteins involved in uptake, excretion, and intracellular storage or trafficking of zinc. These proteins are metallothioneins (MTs) and transmembrane transporters, which include ZIP and cation diffusion facilitator (CDF) families [20,21]. MTs belong to a family of low molecular weight, cysteine-rich intracellular proteins that bind transition metals, including zinc and cadmium [22], and their biological roles include the detoxification of harmful metals and the homeostasis of essential metals [23]. The most prominent characteristic of MT is its inducibility not only by metals such as zinc, cadmium, and copper but also by various factors such as hormones, cytokines, organic chemicals, starvation, and physical stress [24]. The roles of MT and zinc transporters in zinc homeostasis in liver after PS exposure have been poorly understood.

The aim of present study is to assess the effects of repeated PS on the zinc metabolism and related mechanism involved in the homeostasis of zinc in liver.

## Methods

### Animals

Fifty male Sprague-Dawley rats (Shanghai-BK Co., Ltd. Shanghai, China), 8 weeks old, weighing 200 ± 20 g, were housed individually in cages at a temperature of 25 ± 1°C, a humidity of 55 ± 5% in a 12-h light/dark cycle, and were given normal chow and free access to water. All animal treatments were strictly in accordance with international ethical guidelines and the National Institutes of Health Guide concerning the Care and Use of Laboratory Animals, and the experiments were carried out with the approval of the Committee of Experimental Animal Administration of the University. The zinc content in diet was 50 mg/kg. After 3 d adaption, the rats were divided into three groups randomly: control group, PS group and foot shock group. The control and PS groups were subdivided into two subgroups: 7 d group and 14 d group (10 rats in every subgroup).

### PS exposure

PS model was created in rats by a communication box as described previously [14,16-18]. Briefly, a communication box was divided into room A and room B with a transparent acrylic board. Room A included ten little rooms with a plastic board-covered floor and room B included ten little rooms with a metal grid-exposed floor for electric insulation. Rats in room B were randomly given electrical shock (0.6 mA for 1 s) for 30 min (60 times) through the floor and exhibited nociceptive stimulation-evoked responses, such as jumping up, defecation, and crying; rats in room A were only exposed to the responses of rats in room B to establish PS model. PS was given to rats for 30 min every morning (10:00–10:30) for 14 days. At the end of the exposure, the rats were kept in the cages for another 4 min before they were taken out. Animals in the control group were only kept in the cages for 4 min without receiving any stress. During the experiment, weight was monitored.

### Tissues preparation

At the end of PS exposure, all rats were deeply anesthetized by intraperitoneal injection of 7% chloral hydrate. Blood samples were collected from the heart followed by centrifuging at 3,000 × g for 15 min, and the supernatants were obtained and stored at -80°C for further determination. Then the rats were perfused through the left cardiac ventricle with ice-cold phosphate-buffered saline (pH 7.4). The liver was quickly removed and snaps frozen in liquid nitrogen, and kept in a -80°C freezer till use.

### Corticosterone (CORT) and MT measurement

CORT in serum and MT in liver were analyzed using commercially available ELISA kits (R&D Systems, Inc., USA). Liver was homogenized and lysed for ELISA. Total protein concentration was determined by the method described by Lowry et al. with bovine serum albumin as the standard.

### Determination of zinc contents in liver and serum

Each liver sample was weighed and wet-acid digested with a concentrated nitric/perchloric acid mixture (4:1 ratio) for 24 h. An aliquot of each sample was analyzed using a zinc flame or graphite atomic absorption spectrophotometer (Z-8100, Hitachi, Tokyo, Japan). Serum zinc concentration was also measured using the blood obtained from rats in the same manner. The zinc contents were expressed as micrograms zinc per gram tissue or milliliter serum.

### Quantification of ZIP14 mRNA levels

Total RNA was extracted using Trizol reagent (Invitrogen) following the manufacturers' recommendations. All RNA samples were treated with ten units of RNAse-free DNAse I (Promega) according to manufacturer's recommendations, and 1 μg RNA was then reverse transcribed into cDNA with 2.5 pmol oligo(dT)_18_ primer and 5 U avian myeloblastosis virus reverse transcriptase XL (TaKaRa) in 25 μl reaction mixture at 42°C for 40 min according to manufacturer's recommendations. The ZIP14 mRNA level was quantified by the real-time PCR technique using a Thermal Cycler Dice Real Time System (Rotor-gene 3000) with rat ZIP14 primer Forward: 5′-AAGGAAAATGAGCAGACAGAGG-3′, Reverse: 5′-AGAAAAGGTAGAAACCCCCAAA-3′; GAPDH primer Forward: 5′-GGCTCTCTGCTCCTCCCTGTTCTAG -3′, Reverse: 5′-CGTCCGATACGGCCAAATCCGT -3′ and a SYBR Premix Ex Taq kit (Takara).

### Statistical analysis

All results were expressed as means ± SD. Statistical analysis was carried out by using SPSS 11.0. One-way ANOVA, correcting for differences in sample variance, was used to determine whether differences were statistically significant in groups. A *P* value less than 0.05 was a considered statistically significant difference.

## Results

### Body weight gain

**Figure 1:**
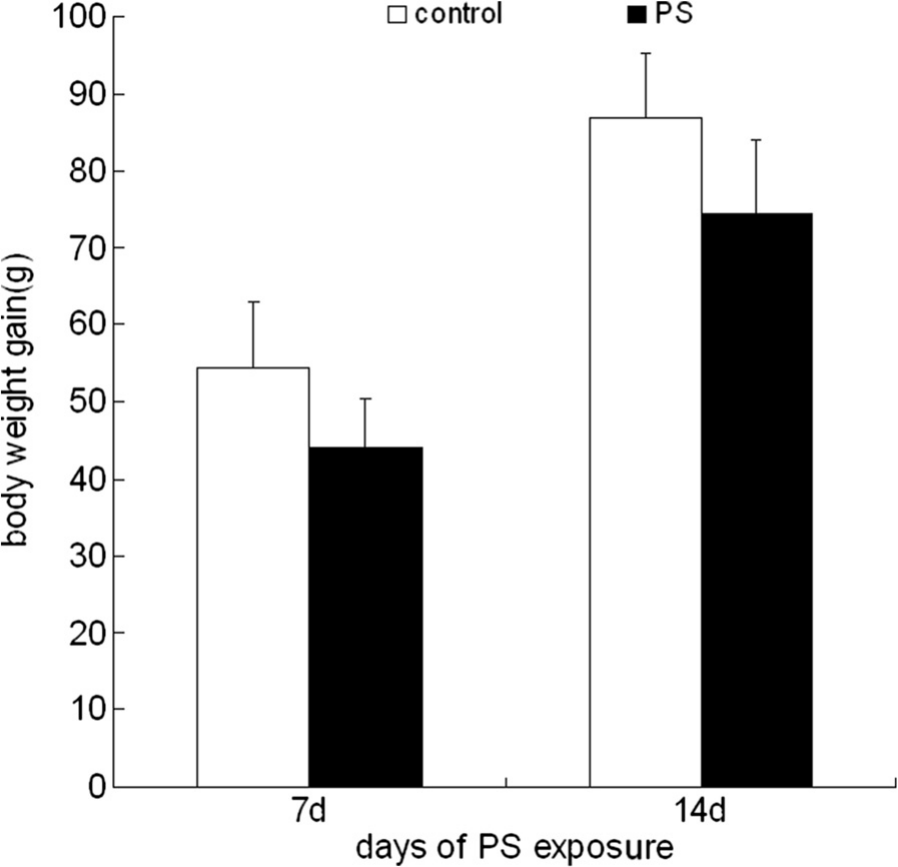
**Body weight gain in rats after 7 d and 14 d PS exposure.** Values were expressed as means ± SD, n = 10.

The body weight of rats was monitored after 7 d and 14 d PS exposure. No significant differences have been observed for the body weight gain between control group and PS group after 7 d and 14 d PS exposure (Figure 1).

### CORT content in serum

**Figure 2:**
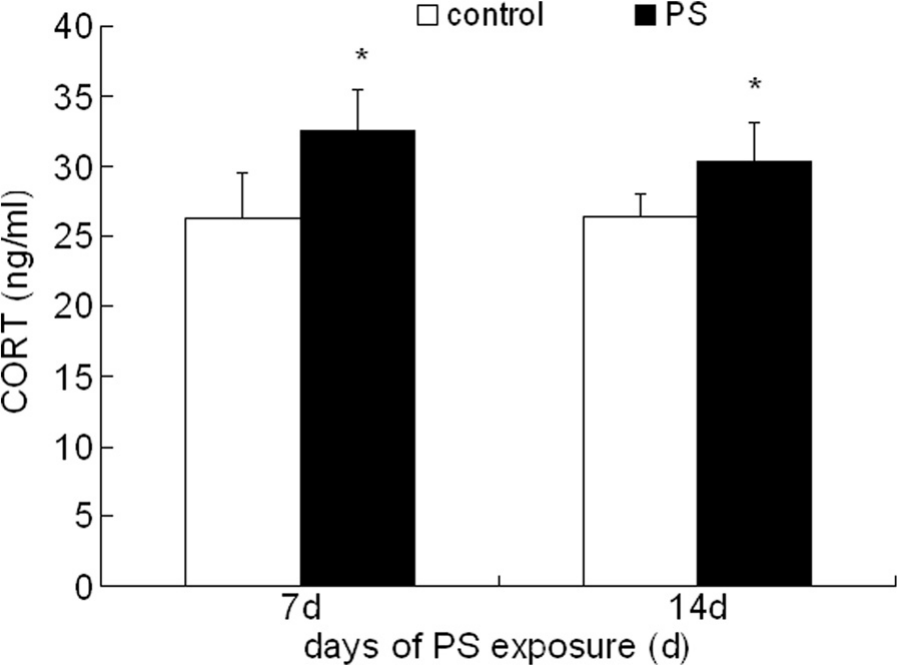
**CORT levels in rat after 7 d and 14 d PS exposure.** Values were expressed as means ± SD, n = 10. * *P* < 0.05 compared to control group.

As shown in Figure 2, the level of CORT in rat serum increased significantly after PS treatment compared to control group. There is no significant difference in CORT level between 7 d and 14 d PS groups.

### Zinc contents in serum and liver

**Figure 3:**
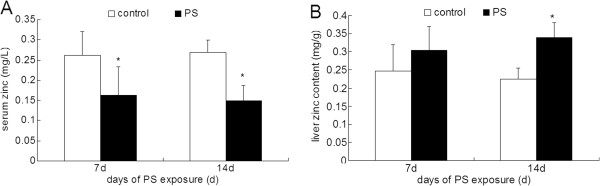
**Zinc in serum (A) and liver (B) in rat after 7 d and 14 d PS exposure.** The zinc contents were expressed as micrograms zinc per gram wet tissue or milliliter serum. Values were expressed as means ± SD, n = 10. * *P* < 0.05 compared to control group.

PS significantly decreased the zinc concentration in the serum after 7 d and 14 d PS exposure (Figure 3A). Meanwhile, zinc content in liver was elevated after 14 d PS exposure, while elevation of zinc content in the liver after 7 d PS exposure was not significant (Figure 3B).

### MT level in liver

**Figure 4:**
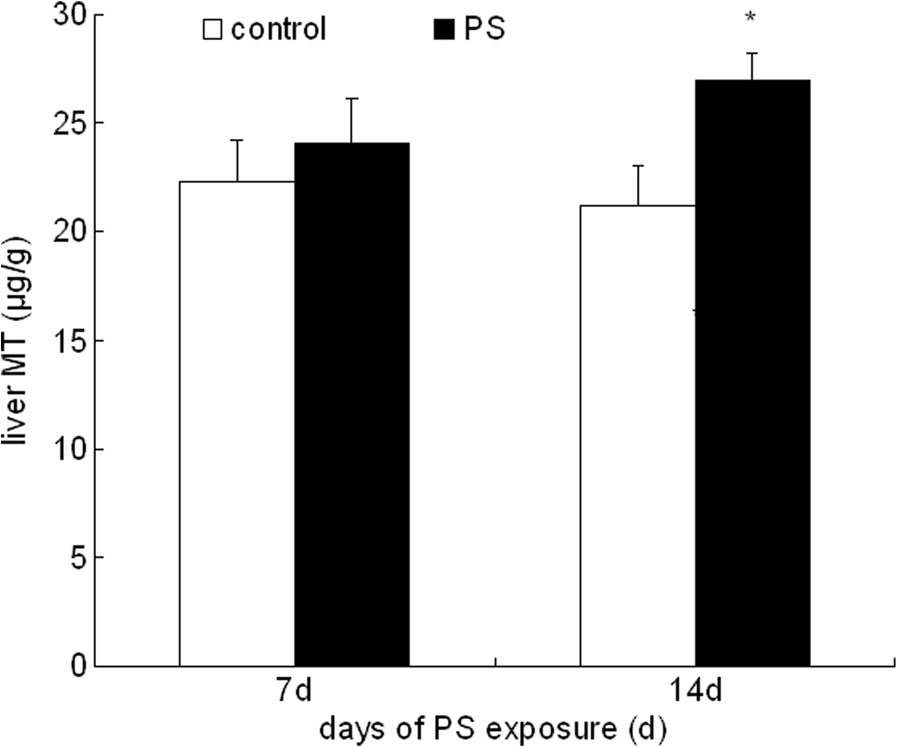
**MT content in liver after 7 d and 14 d PS exposure.** Values were expressed as means ± SD, n = 10. * *P* < 0.05 compared to control group.

The MT level was coincident with the zinc content in liver after PS. MT content in liver was elevated after 14 d PS exposure, but no significant change was observed in the 7 d PS exposure group as shown in Figure 4.

### ZIP14 mRNA expression in liver

**Figure 5:**
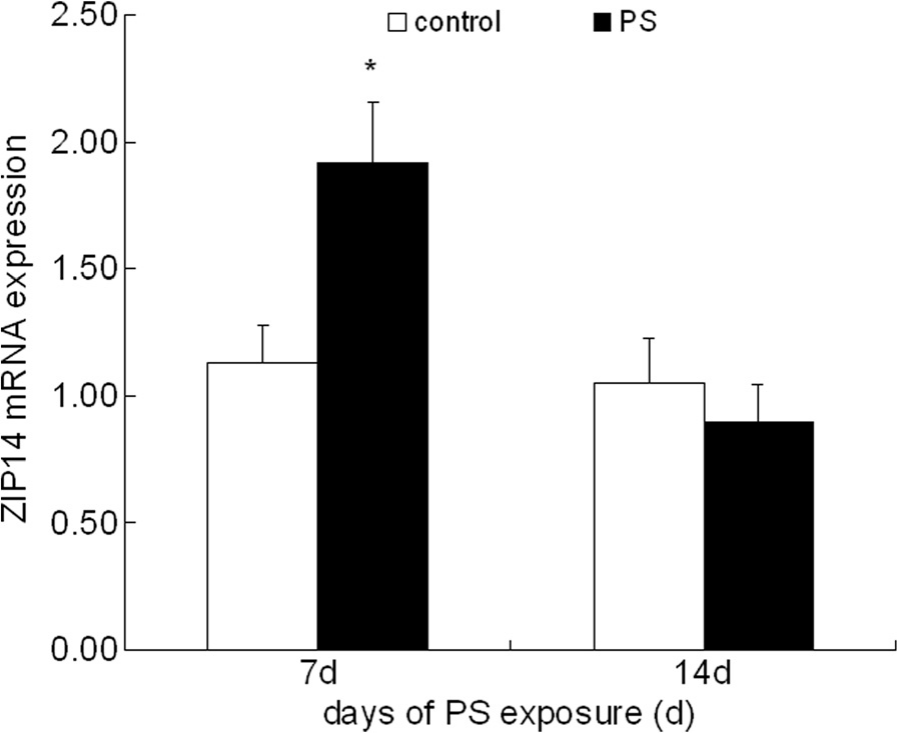
**ZIP14 mRNA expression in liver after 7 d and 14 d PS exposure.** GAPDH was used as the RNA control. Values were expressed as means ± SD, n = 10. * *P* < 0.05 compared to control group.

Time-dependent expression of Zip14 mRNA in the liver under PS was examined by quantitative RT-PCR. Zip14 mRNA was expressed markedly at 7 d after the onset of PS (Figure 5), while Zip14 mRNA expression in the liver after 14 d PS exposure reached normal level compared with control group.

## Discussion

Multiple biological macromolecules and physical cell events involve zinc as a structural component or as a major regulator. As a consequence, zinc is a metal that is essential for several aspects of normal development and health. The occurrence of zinc deficiency and the prevention of oxidative damage by zinc supplementation have been observed in various cells and tissues. A decrease in zinc availability is associated with an increase in cellular oxidants, alterations in the antioxidant defense components, and increased oxidation parameters [25,26]. Zinc has a variety of effects on biological activities that might explain this hepatoprotective action. These are the following: (1) Zinc stabilizes membranes and inhibits lipid peroxidation; (2) Zinc induces hepatic MT, which is rich in SH groups and binds certain toxic metals; (3) Zinc is required for P450 activity, which is important in toxic drug metabolism; (4) Zinc improves the protein synthesis function of liver; and (5) the hepatoprotective action of zinc administration against oxidative stress was imposed through the regulation of the metabolism of superoxide dismutase, MT and reduced glutathione [27,28]. Liver disease, especially alcoholic liver disease, has been associated with hypozincemia and zinc deficiency [29]. Besides, liver cirrhosis is frequently accompanied by malnutrition and zinc deficiency, a state closely associated with the development of ascites [30]. The administration of branched amino acids and zinc leads to an increase of zinc and albumin levels in the serum [31] and an improvement in nutrition state [32]. The substrates albumin and zinc stimulate each other reciprocally. Zinc is a very important factor in albumin synthesis and albumin is the primary transport medium for zinc [33]. In present study, we observed decreased serum zinc level and increased liver zinc level after 7 d and 14 d PS exposure. It is well known that zinc absorption is regulated by a number of factors. Zinc intake also could affect zinc absorption in the small intestine. In this study, the standard diet contained 50 mg/kg zinc was given to exclude the factors related to feeding. No significant differences for diet uptake and body weight gain were detected between the control and PS groups. In our previous study, we found that PS could induce the decreased iron absorption, and then hypoferremia occurred [34]. In order to clarify the detail mechanism of hypozincemia induced by PS, further studies should be carried out, which regard effects on zinc absorption in rats treated by PS exposure. Besides, the elevated hepatic zinc concentration in these rats indicated that PS changed zinc distribution, limited the transportation and utilization of zinc. Therefore, we proposed that liver zinc accumulation might be one of the reasons to aggravate hypozincemia in rats after PS exposure. Furthermore, the results support the use of supplementation of zinc and branched amino acids in liver cirrhosis patients with ascites.

Communication box is a common model to investigate the physical and physiological changes under PS [35,36]. Without direct physical stress, the box can produce an experimental anxiety based on intraspecies emotion. In our study, serum CORT increased significantly after PS, which indicated that the emotional responses to foot shock activated the hypothalamic-pituitary-adrenal (HPA) axis in PS rats. Exposure to stress induces the synthesis of MT, which is low-molecular-weight proteins, rich in cysteine which confers them with a high capacity to bind heavy metal ions (zinc, cadmium, copper, silver, mercury, etc.) in biological systems [24]. This induction is well correlated with the increase in stress-induced serum glucocorticoids as shown in present study [37]. Moreover, adrenalectomy (ADX) had a general decreasing effect on basal MT level [38]. The promoter region glucocorticoid response elements, metal response elements, the antioxidant response elements, and the elements activated by signal transducers and activators of transcription are present in the upstream of the chromosome that codes for MT gene, and are known to be involved in the expression of MT induced by glucocorticoids, zinc, activated oxygen, and cytokines [39]. Zinc is a metal bound to MT. The decrease in serum zinc after exposure to PS may due partially to the significant increase in MT synthesis in the liver which was observed in present study, because zinc absorbed from the small intestine is transported to the liver and then redistributed to other tissues [40,41]. Cell zinc uptake is facilitated by MT synthesis, which might be one of causes decreasing extracelluar zinc concentration. The present study suggests that exposure to PS decreases extracelluar zinc level through glucocorticoid-mediated MT synthesis. The relationship between stress and zinc signaling is an important issue.

Maintaining cellular zinc homeostasis is of crucial importance for the functions and effects of zinc [42]. Zinc is a trace nutrient indispensable for organisms and its homeostasis is mostly regulated by zinc transporters and MT. Known eukaryotic zinc transporters are largely classified into two families, the ZIP and ZnT families, which are designated as “SLC39” and “SLC30” respectively. ZIP members facilitate zinc influx into the cytosol, while ZnT members facilitate its efflux from the cytosol [43,44]. Zinc in the liver is stored in two forms: one from which it can be mobilized rapidly, and one with only slow mobilization. The regulatory process depends almost completely on hormonal control by insulin, glucagon and the glucocorticoids. Depending on the metabolic situation, these substances trigger a transient dysregulation of zinc metabolism with subsequent serum zinc deficiency and redistribution in various tissues, particularly in liver. Mediator substances have similar effects [45]. There are tight interactions between MT, an acute–phase protein, interleukin-6 (IL-6) and zinc. IL-6, the most important proinflammatory cytokine regulating the acute–phase protein production regulates ZIP14 in liver and contributes to the zinc deficiency of acute–phase response [46]. ZIP14, one of the ZIP family transporters, is involved in zinc uptake in cells. ZIP14 was localized in the plasma membrane of hepatocytes. The *in vivo* and *in vitro* experiments demonstrated that ZIP14 expression was up-regulated by turpentine-induced inflammation and lipopolysaccharide administration through IL-6, and that this zinc transporter most likely plays a major role in the mechanism responsible for hepatic zinc accumulation and hypozincemia that accompanied the acute-phase response to inflammation and infection. In addition, recent experiments suggest that signaling pathways activated by nitric oxide are factors in the upregulation of ZIP14, which in turn mediates hepatic zinc accumulation and hypozincemia during inflammation and sepsis [47]. The physiological roles of ZIP14 are still speculative. In present study, ZIP14 mRNA expression was markedly up-regulated after 7 d PS exposure. IL-6 increased by PS mentioned in our previous study. These results suggest that ZIP14, an IL-6 responsive zinc transporter which facilitates extracelluar zinc into cytosol, was induced through IL-6 because of PS exposure, followed by the increase in the zinc concentration in the liver. After 14 d PS exposure, ZIP14 reached normal level, which was the result of zinc accumulation in liver induced by PS.

## Conclusions

Collectively, PS exposure strongly induced a reduction in the serum zinc and liver zinc accumulation, which were correlated with up-regulation of ZIP14 mRNA through IL-6 and MT protein through CORT. However, the exact mechanism of these interactions needs to be further investigated.

## Competing interests

The authors declare that they have no competing interests.

## Authors’ contributions

XT carried out the animal model and the measurement of zinc apparent absorption studies. YZ and YL carried out the ELISA measurement and drafted the manuscript. ZS carried out atomic absorption spectrophotometry. LT and XD carried out the real-time PCR and statistical analysis. JQ and HS edited and revised the manuscript and organized the study. All authors read and approved the final manuscript.

## Pre-publication history

The pre-publication history for this paper can be accessed here:

http://www.biomedcentral.com/1471-230X/14/32/prepub

## References

[B1] Tuerk MJ Fazel N Zinc deficiency Curr Opin Gastroenterol 2009 25 136 143 10.1097/MOG.0b013e328321b395 19528881

[B2] Krebs NE Hambidge KM Zinc metabolism and homeostasis: the application of tracer techniques to human zinc physiology Biometals 2001 14 397 412 10.1023/A:1012942409274 11831468

[B3] Stamoulis I Kouraklis G Theocharis S Zinc and the liver: an active interaction Dig Dis Sci 2007 52 1595 1612 10.1007/s10620-006-9462-0 17415640

[B4] Grüngreiff K Reinhold D Liver cirrhosis and "liver" diabetes mellitus are linked by zinc deficiency Med Hypotheses 2005 64 316 317 10.1016/j.mehy.2004.04.030 15607563

[B5] Liu J Kershaw WC Klaassen CD Protective effects of zinc on cultured rat primary hepatocytes to metals with low affinity for metallothionein J Toxicol Environ Health 1992 35 51 62 10.1080/15287399209531593 1728665

[B6] DiSilvestro RA Carlson GP Effects of mild zinc deficiency, plus or minus acute phase response, on CCl4 hepatotoxicity Free Radic Biol Med 1994 16 57 61 10.1016/0891-5849(94)90243-7 8299997

[B7] Afonne OJ Orisakwe OE Ndubuka GI Akumka DD Ilondu N Zinc protection of mercury induced hepatic toxicity in mice Biol Pharm Bull 2000 23 305 308 10.1248/bpb.23.305 10726883

[B8] Mohammad MK Zhou Z Cave M Barve A McClain CJ Zinc and liver disease Nutr Clin Pract 2012 27 8 20 10.1177/0884533611433534 22307488 PMC6027651

[B9] Wales JK Does psychological stress cause diabetes? Diabet Med 1995 12 109 112 10.1111/j.1464-5491.1995.tb00439.x 7743755

[B10] Sherwood A Allen MT Obrist PA Langer AW Evaluation of beta-adrenergic influences on cardiovascular and metabolic adjustments to physical and psychological stress Psychophysiology 2007 23 89 104 3003780 10.1111/j.1469-8986.1986.tb00602.x

[B11] Epel ES Psychological and metabolic stress: a recipe for accelerated cellular aging? Hormones (Athens) 2009 8 7 22 10.14310/horm.2002.1217 19269917

[B12] Valko M Rhodes CJ Moncol J Izakovic M Mazur M Free radicals, metals and antioxidants in oxidative stress-induced cancer Chem Biol Interact 2006 160 1 40 10.1016/j.cbi.2005.12.009 16430879

[B13] Buijsse B Feskens EJ Moschandreas J Jansen EH Jacobs DR Jr Kafatos A Kok FJ Kromhout D Oxidative stress, and iron and antioxidant status in elderly men: differences between the Mediterranean south (Crete) and northern Europe (Zutphen) Eur J Cardiovasc Prev Rehabil 2007 14 495 500 10.1097/HJR.0b013e3280111e41 17667637

[B14] Wang L Wang W Zhao M Ma L Li M Psychological stress induces dysregulation of iron metabolism in rat brain Neuroscience 2008 155 24 30 10.1016/j.neuroscience.2008.03.091 18555617

[B15] Scarpellini F Sbracia M Scarpellini L Psychological stress and lipoperoxidation in miscarriage Ann N Y Acad Sci 1994 709 210 213 10.1111/j.1749-6632.1994.tb30404.x 8154708

[B16] Zhao M Chen J Wang W Wang L Ma L Shen H Li M Psychological stress induces hypoferremia through the IL-6-hepcidin axis in rats Biochem Biophys Res Commun 2008 373 90 93 10.1016/j.bbrc.2008.05.166 18541141

[B17] Wei C Zhou J Huang X Li M Effects of psychological stress on serum iron and erythropoiesis Int J Hematol 2008 88 52 56 10.1007/s12185-008-0105-4 18543064

[B18] Li Y Zheng Y Qian J Chen X Shen Z Tao L Li H Qin H Li M Shen H Preventive effects of zinc against psychological stress-induced iron dyshomeostasis, erythropoiesis inhibition, and oxidative stress status in rats Biol Trace Elem Res 2012 147 285 291 10.1007/s12011-011-9319-z 22274754

[B19] Ohashi T Matsui T Chujo M Nagao M Restraint stress up-regulates expression of zinc transporter Zip14 mRNA in mouse liver Cytotechnology 2008 57 181 185 10.1007/s10616-008-9148-x 19003164 PMC2553665

[B20] Eide D Molecular biology of iron and zinc uptake in eukaryotes Curr Opin Cell Biol 1997 9 573 577 10.1016/S0955-0674(97)80036-1 9263657

[B21] Eide DJ The SLC39 family of metal ion transporters Pflugers Arch 2004 447 796 800 10.1007/s00424-003-1074-3 12748861

[B22] Kagi JH Valee BL Metallothionein: a cadmium- and zinc-containing protein from equine renal cortex J Biol Chem 1960 235 3460 3465 13750713

[B23] Cherian MG Howell SB Imura N Klaassen CD Koropatnick J Lazo JS Waalkes MP Role of metallothionein in carcinogenesis Toxicol Appl Pharmacol 1994 126 1 5 10.1006/taap.1994.1083 8184419

[B24] Sutherland DE Stillman MJ The "magic numbers" of metallothionein Metallomics 2011 3 444 463 10.1039/c0mt00102c 21409206

[B25] Song Y Leonard SW Traber MG Ho E Zinc deficiency affects DNA damage, oxidative stress, antioxidant defenses, and DNA repair in rats J Nutr 2009 139 1626 1631 10.3945/jn.109.106369 19625698 PMC3151020

[B26] Varghese J Faith M Jacob M Zinc prevents indomethacin-induced renal damage in rats by ameliorating oxidative stress and mitochondrial dysfunction Eur J Pharmacol 2009 614 114 121 10.1016/j.ejphar.2009.04.053 19445918

[B27] Tupe RS Tupe SG Tarwadi KV Agte VV Effect of different dietary zinc levels on hepatic antioxidant and micronutrients indices under oxidative stress conditions Metabolism 2010 59 1603 1611 10.1016/j.metabol.2010.02.020 20359724

[B28] Oteiza PI Zinc and the modulation of redox homeostasis Free Radic Biol Med 2012 53 1748 1759 10.1016/j.freeradbiomed.2012.08.568 22960578 PMC3506432

[B29] Kang YJ Zhou Z Zinc prevention and treatment of alcoholic liver disease Mol Aspects Med 2005 26 391 404 10.1016/j.mam.2005.07.002 16099027

[B30] Kuiper JJ De MA RA Van Buhren HR Management of ascites and associated complications in patients with cirrhosis Aliment Pharmacol Ther 2007 26 183 193 18081661 10.1111/j.1365-2036.2007.03482.x

[B31] Grüngreiff K Branched chain amino acids ( BCAAs) and zinc in treatment of ascites in liver cirrhosis Hepatol Int 2011 5 345

[B32] Nishitani S Takehana K Pharmacological activities of branched amino acids: Augmentation of albumin synthesis in liver and improvement in skeletal muscle Hepatol Res 2004 30 S19 S24 10.1016/j.hepres.2004.08.006 15607134

[B33] Lu J Stewart AJ Sadler PJ Pinheiro TJ Blindauer CA Albumin as a zinc carrier: properties of its high-affinity zinc-binding site Biochem Soc Trans 2008 36 1317 1321 10.1042/BST0361317 19021548

[B34] Chen J Shen H Chen C Wang WY Yu SY Zhao M Li M The effect of psychological stress on iron absorption in rats BMC Gastroenterol 2009 9 83 10.1186/1471-230X-9-83 19912618 PMC2783024

[B35] Endo Y Shiraki K Behavior and body temperature in rats following chronic foot shock or psychological-stress exposure Physiol Behav 2000 71 263 268 10.1016/S0031-9384(00)00339-5 11150557

[B36] Endo Y Yamauchi K Fueta Y Irie M Changes of body temperature and plasma corticosterone level in rats during psychological stress induced by the communication box Med Sci Monit 2001 7 1161 1165 11687724

[B37] Jacob ST Ghoshal K Sheridan JF Induction of metallothionein by stress and its molecular mechanisms Gene Expr 1999 7 301 310 10440231 PMC6174668

[B38] Hidalgo J Belloso E Hernandez J Gasull T Molinero A Role of glucocorticoids on rat brain metallothionein-I and -III response to stress Stress 1997 1 231 240 10.3109/10253899709013743 9787247

[B39] Sato M Kondoh M Recent studies on metallothionein: protection against toxicity of heavy metals and oxygen free radicals Tohoku J Exp Med 2002 196 9 22 10.1620/tjem.196.9 12498322

[B40] Cousins RJ Toward a molecular understanding of zinc metabolism Clin Physiol Biochem 1986 4 20 30 2420502

[B41] Tamano H Enomoto S Igasaki E Oku N Itoh N Kimura T Tanaka K Takeda A Hepatic zinc response via metallothionein induction after tumor transplantation Biochem Biophys Res Commun 2000 270 1140 1143 10.1006/bbrc.2000.2557 10772964

[B42] Maret W Zinc biochemistry: from a single zinc enzyme to a key element of life Adv Nutr 2013 4 82 91 10.3945/an.112.003038 23319127 PMC3648744

[B43] Huang L Tepaamorndech S The SLC30 family of zinc transporters - A review of current understanding of their biological and pathophysiological roles Mol Asp Med 2013 34 548 560 10.1016/j.mam.2012.05.008 23506888

[B44] Jeong J Eide D The SLC39 family of zinc transporters Mol Asp Med 2013 34 612 619 10.1016/j.mam.2012.05.011 PMC3602797 23506894

[B45] Grüngreiff K Reinhold D Rink L Zinc and Liver Zinc in Human Health 2011 Amsterdam: IOS Press 473 492

[B46] Liuzzi JP Lichten LA Rivera S Blanchard RK Aydemir TB Knutson MD Ganz T Cousins RJ Interleukin-6 regulates the zinc transporter Zip14 in liver and contributes to the hypozincemia of the acute-phase response Proc Natl Acad Sci USA 2005 102 6843 6848 10.1073/pnas.0502257102 15863613 PMC1100791

[B47] Lichten LA Liuzzi JP Cousins RJ Interleukin-1beta contributes via nitric oxide to the upregulation and functional activity of the zinc transporter Zip14 (Slc39a14) in murine hepatocytes Am J Physiol Gastrointest Liver Physiol 2009 296 G860 G867 10.1152/ajpgi.90676.2008 19179618 PMC2670674

